# Macropinocytosis: A Metabolic Adaptation to Nutrient Stress in Cancer

**DOI:** 10.3389/fendo.2017.00261

**Published:** 2017-09-29

**Authors:** Maria Victoria Recouvreux, Cosimo Commisso

**Affiliations:** ^1^Tumor Initiation and Maintenance Program, NCI-Designated Cancer Center, Sanford Burnham Prebys Medical Discovery Institute, La Jolla, CA, United States

**Keywords:** macropinocytosis, Ras, growth factors, nutrient uptake, cancer metabolism

## Abstract

Oncogenic mutations, such as Ras mutations, drive not only enhanced proliferation but also the metabolic adaptations that confer to cancer cells the ability to sustain cell growth in a harsh tumor microenvironment. These adaptations might represent metabolic vulnerabilities that can be exploited to develop novel and more efficient cancer therapies. Macropinocytosis is an evolutionarily conserved endocytic pathway that permits the internalization of extracellular fluid *via* large endocytic vesicles known as macropinosomes. Recently, macropinocytosis has been determined to function as a nutrient-scavenging pathway in Ras-driven cancer cells. Macropinocytic uptake of extracellular proteins, and their further degradation within endolysosomes, provides the much-needed amino acids that fuel cancer cell metabolism and tumor growth. Here, we review the molecular mechanisms that govern the process of macropinocytosis, as well as discuss recent work that provides evidence of the important role of macropinocytosis as a nutrient supply pathway in cancer cells.

## Introduction

Sustained rapid proliferation represents a major metabolic hurdle for cancer cells. The challenge lies in balancing the towering energy and nutrient demands required for biomass production with the harsh nutrient-depleted conditions of the tumor microenvironment. Hence, it is not surprising that tumors have evolved the capacity to employ the same oncogenic signaling pathways [e.g., RAS, MYC, PI3-kinase (PI3K)] that trigger aberrant growth to also control the metabolic rewiring that is necessary to adapt to a nutrient-deprived ecosystem (1).

Metabolic reprogramming is now recognized as one of the hallmarks of cancer cells (2) and the topic has become of increasing interest in recent years. Importantly, a better understanding of the molecular mechanisms and metabolic adaptations that confer growth and survival advantages to cancer cells could lead to the discovery of novel therapeutic opportunities. One of the many adaptive strategies that cancer cells use to fulfill their metabolic demands is the ability to exploit alternative nutrient acquisition pathways (3). Among them, macropinocytosis is an evolutionarily conserved form of bulk endocytosis by which cells incorporate extracellular fluid into large, irregularly shaped vesicles called macropinosomes (4). Macropinocytosis was first observed microscopically in malignant cells in the 1930s (5), and since then, it has been extensively studied in different cell types and in varying contexts. For example, the amoeboid organism *Dictyostelium discoideum* utilizes macropinocytic uptake to engulf fluid and nutrients during axenic growth (6). In antigen presentation that occurs in the mammalian immune system, macrophages and dendritic cells employ macropinocytosis to internalize and process extracellular antigenic proteins (7). Macropinocytosis can occur at basal rates, as it occurs spontaneously in many cells, or it can be dramatically induced by receptor tyrosine kinase (RTK) activation or by oncogenes such as Ras (8) and v-Src (9). Although having been observed in transformed cells for nearly 30 years, the biological relevance of macropinocytosis in cancer has only recently been elucidated (10). In cancer cells harboring oncogenic Ras mutations, macropinocytosis serves as a nutrient uptake pathway by which extracellular protein is internalized and degraded to supply the much-needed amino acids that support cellular growth (10–12). This novel function of macropinocytosis as a feeding mechanism in tumors has provided a new perspective for cancer metabolism research and has positioned this ancient mechanism as a promising target for therapeutic intervention.

In this review, we aim to summarize the mechanisms that govern macropinocytosis regulation in cancer cells, as well as to provide the latest findings supporting its important role as a nutrient supply pathway that enables tumor cell proliferation and survival.

## Regulation of Macropinocytosis in Cancer Cells

Macropinocytosis is a clathrin-independent endocytic process driven by actin. In contrast to the closely related phagocytosis pathway, macropinocytic uptake is non-selective and not controlled by its cargo (13). Other distinctive features that define macropinocytosis are the fact that it can be stimulated by growth factors, and that it is suppressed by ion exchange inhibitors such as amiloride and its derivatives, which specifically inhibit macropinocytosis as opposed to other endocytic pathways (4, 14). Another definitive property is that macropinosomes are larger than other endocytic vesicles and can be specifically labeled by high molecular weight dextrans (15), which are incorporated into discrete vesicles larger than 0.2 µm in diameter.

Macropinocytosis is intimately linked to actin cytoskeleton dynamics. Protrusions of the plasma membrane, known as membrane ruffles, are formed *via* actin polymerization. Nascent macropinosomes arise at the cell surface of ruffling cells when these membrane protrusions spontaneously form cup-shaped ruffles that close, leading to fission of the nascent macropinosome from the plasma membrane and the internalization of extracellular fluid (13). Two types of membrane ruffles have been described and both can lead to macropinocytosis: planar ruffles, which are derived from the cell edges, and circular dorsal ruffles, which occur at the apical cell surface (16).

Both membrane ruffling and macropinocytosis depend heavily on actin cytoskeleton remodeling, as evidenced by their complete abrogation by inhibitors that disrupt actin polymerization, like cytochalasin D (17, 18). Moreover, many key regulators of actin polymerization, such as members of the Ras superfamily of small guanosine triphosphatases (GTPases), Ras, Rac, Cdc42, Arf6, and Rab5, among others, have been associated with ruffle formation and macropinocytic activity (19). Additionally, membrane phospholipids, in particular phosphatidylinositol (PI), PI4P, PI5P, PI(4,5)P_2_, and PI(3,4,5)P_3_, and the phospholipid kinases and phosphatases that interconvert them, are important players in the spatiotemporal regulation of macropinocytosis (13). For instance, inhibition of PI3K by either wortmannin or LY294002 has been shown to abolish macropinocytosis in several cell types including cancer cells, fibroblasts, and macrophages (18, 20, 21). Stage-specific enrichment of each type of PI during macropinosome maturation allows for the sequential recruitment and activation of specific enzymes and adapter proteins, including small GTPases and other proteins necessary for actin polymerization and membrane trafficking, such as Scar/Wave, Wasp, and Arp2/3 complexes, as well as sorting nexins (22–24).

**Figure 1:**
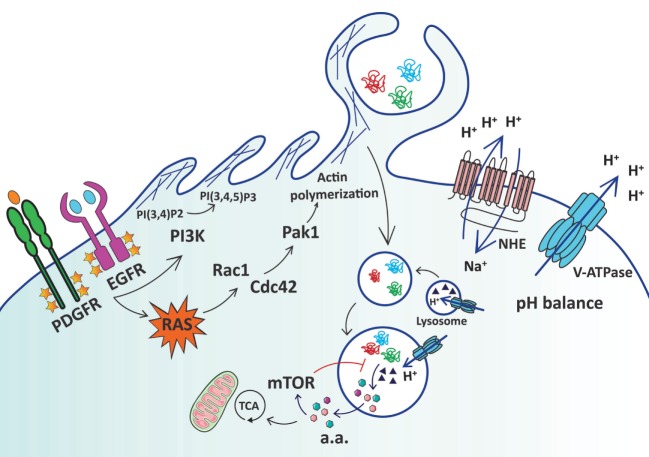
Schematic representation of extracellular protein uptake *via* macropinocytosis in cancer cells. Ras activation, either by growth factor stimulation or through oncogenic mutation, leads to increased membrane ruffling and macropinocytosis *via* activation of Rac1 and Cdc42, which in turn stimulate p21-activated kinase 1 (Pak1) to induce actin polymerization. Activation of Rac1 and Cdc42 is sensitive to changes in submembranous pH, and the activity of Na+/H+ exchangers (NHEs) and vacuolar H+-ATPase (V-ATPases) is crucial to maintaining pH homeostasis. Conversion of membrane phosphoinositides by PI3-kinase (PI3K) is also necessary for macropinocytosis. Macropinosomes containing extracellular proteins such as albumin and collagen are internalized and subsequently fuse with lysosomes. Lysosomal proteases (▲) allow the catabolism of extracellular proteins into free amino acids (a.a.) that can fuel the TCA cycle to promote cell growth and survival. mTORC1 finely regulates the utilization of extracellular protein-derived amino acids by inhibiting macropinocytosed protein catabolism when free a.a. are abundant. Yellow stars represent phosphorylation of growth factor receptors.

These orchestrated rearrangements of the actin cytoskeleton, as well as the production and turnover of phospholipids necessary for macropinocytosis, can be initiated by growth factor-dependent activation of RTKs (Figure 1). Induction of macropinocytosis by growth factors, such as epidermal growth factor (EGF), platelet-derived growth factor (PDGF), and macrophage colony-stimulating factor, has been well studied and relies on the capacity of growth factors to stimulate membrane ruffling through the activation of the small GTPases Ras and Rac (14, 25–27). Several studies have demonstrated that Ras activation, either by growth factor stimulation or through oncogenic mutation, leads to increased membrane ruffling and macropinocytosis. First observations by Bar-Sagi and Feramisco showed that microinjection of oncogenic Hras into rat embryo fibroblasts rapidly induced ruffles and fluid-phase uptake of high molecular weight dextran (8). Similarly, Kras-transformed Rat-1 fibroblasts showed increased macropinocytosis that was dependent on PI3K and phospholipase C activity (18). More recently, it was shown that human bladder and pancreatic cancer cells that harbor oncogenic *HRAS* or *KRAS* mutations, respectively, display an enhancement of macropinocytosis relative to cancer cells of the same tissue type that express wild-type *HRAS* or *KRAS* (10). Furthermore, macropinocytic activity was also observed *in vivo* in a *Kras*-mutant mouse model of pancreatic ductal adenocarcinoma (PDAC) (10, 28), as well as in human primary PDAC specimens (11).

Ras activation leads to the stimulation of a plethora of different signal transduction pathways, including Rac, Cdc42, PI3K, and Raf/Erk activation. Activation of Rac by Ras has been shown to have an important role in inducing both membrane ruffling and macropinocytosis in different cell types (26). Both Rac1 transient activation and subsequent deactivation are required for complete closure and maturation of macropinosomes (29). Interestingly, Rac1 and Cdc42 are necessary and sufficient to induce macropinocytosis uptake in bladder cancer cells, as demonstrated by employing dominant negative and constitutively active forms of these small GTPases (30). In addition, it was recently demonstrated that Dock1, a Rac-specific guanine nucleotide exchange factor, is required for oncogenic Ras-induced macropinocytosis in several cancer cells (31).

Rac and Cdc42 can activate actin polymerization *via* p21-activated kinase 1 (Pak1), which has been shown to co-localize with macropinosomes in 3T3 fibroblasts, and can drive macropinocytosis through circular dorsal ruffles when expressed in a constitutively active form (32, 33). Pak1 activity is also important for closure of macropinocytic cups through phosphorylation of CtBP1/BARS, a protein involved in membrane fission in the context of EGF-induced macropinocytosis (34). Signaling through other members of the Rab family of small GTPases, such as Rab5 and its effector Rabankyrin-5, have been shown to play a role in macropinosome formation and maturation. Specifically, both have been shown to be associated with membrane ruffles in epithelial cells (35, 36) and expression of a Rab5 dominant negative form inhibits PDGF-stimulated circular ruffles in MEFs (37).

While Ras, Rac, and Cdc42 signaling are critical for the early steps of macropinocytosis (i.e., membrane ruffling and formation of macropinocytic cups), PI3K activity seems to be specifically required for macropinosome closure in tumor cells and macrophages (18, 20, 21). Studies in the epithelial carcinoid cell line A431 have shown that PI3K inhibitors do not affect EGFR-induced membrane ruffling, but they inhibit macropinocytosis (21). These studies showed that as the macropinocytic cups close, PI(3,4)P2 is depleted from the cup while PI(3,4,5)P3 production by PI3K increases, and these highly regulated kinetics may be required for coordinated actin remodeling that allows macropinosome closure. It should be noted that these observations may be cell and/or stimuli specific, as other studies have demonstrated that PI3K can control plasma membrane ruffling caused by either oncogenic v-Src expression or PDGF stimulation (18, 38). The regulation of membrane ruffling by PI3K in these contexts may be mediated by PI3K-dependent activation of Rac1 (39).

The fate of macropinosomes after internalization varies depending on the cell type, as they can be recycled to the cell membrane as is the case in A431 cells (40) or they can adopt degradative properties by fusing with lysosomes and undergoing a lysosome-dependent acidification, as is the case in macrophages (41) and Ras-transformed cancer cells (10). Although the mechanisms underlying macropinosome maturation remain to be explored, a switch from Rab5 to Rab7 accumulation on the macropinosome (42) and the recruitment of specific septins to the maturing macropinosome, seem to regulate fusion events with late endosomal/lysosomal compartments (43). Although much work has been done in past years to elucidate the mechanisms that control macropinocytosis, more studies are necessary to further identify the cell- and tissue-specific pathways that regulate this process, especially in cancer cells where it can represent a therapeutic targeting strategy.

## Macropinocytosis and pH Homeostasis

As discussed above, macropinocytic induction arises as a result of the coordinated interactions among small GTPases, actin filaments, and membrane phosphoinositides in restricted areas of the plasma membrane known as membrane ruffles. Because of the electrostatic origin of some of these interactions, they are susceptible to alterations in the charge balance across the plasma membrane. Accordingly, quite soon after the discovery of growth factor-induced macropinocytosis, it was shown that conditions that acidify the cytosol, such as the addition of ${\text{NH}}_{4}^{+}$, or the blockade of the Na^+^/H^+^ exchangers (NHEs) by amiloride, dramatically inhibit macropinocytosis (14). Given that NHE inhibition selectively blocks macropinocytosis, leaving coated vesicles intact, sensitivity to amiloride and its analogs, like 5-(*N*-ethyl-*N*-isopropyl) amiloride (EIPA) and HOE-694, has been used as a distinctive feature of macropinocytosis (4). Nevertheless, the functional association between Na^+^/H^+^ exchange and macropinocytosis and the mechanisms mediating amiloride/EIPA inhibition remained unknown for many years.

Koivusalo et al. described that targeting of NHEs, by amiloride and HOE-694, abrogates EGF-induced macropinocytosis by lowering the submembranous pH (44). Interestingly, these changes in pH (from pH = 7.8 to 6.8) seemed to exclusively affect the recruitment and activation of Rac1 and Cdc42 to membrane ruffles. Consequently, the recruitment of their downstream effectors, Pak1 and Arp2/3, was also abrogated, thus inhibiting ruffle formation without affecting EGFR phosphorylation or PI3K activation. The authors also showed that EGF could stimulate Na^+^/H^+^ exchange with a concomitant alkalinization of the cytoplasm at sites of nascent macropinosome formation. Inhibition of the NHEs resulted in an accumulation of acidic equivalents, which are thought to occur due to a boost of metabolic activity driven by EGF stimulation. The outcome was an overall acidification of the cytoplasm, with a more pronounced effect in the vicinity of the plasma membrane where macropinocytosis was taking place (44). Although this study only addressed NHE inhibition of EGF-induced macropinocytosis in A431 cells, it is likely that similar mechanisms account for amiloride/EIPA inhibition in the setting of other growth factors and in Ras-induced macropinocytosis, where H^+^ accumulation caused by increased metabolic activity and actin polymerization also occurs, especially in cancer cells where the high metabolic rate is well known to promote acidification of the cell and the tumor microenvironment (45–47).

The most commonly used macropinocytosis inhibitors, amiloride and EIPA, broadly target the SLC9A gene family of NHEs, which includes 11 isoforms reported to date (48). Whether specific NHE isoforms differentially regulate macropinocytosis remains an open question. Although NHE1 is highly expressed in several cancer cell lines (49) and is the most widely studied isoform, gene expression analyses indicate that NHE6, 7, and 8 are expressed at levels comparable to NHE1 in PDAC cells (50); therefore, it would be useful to conduct further studies aimed at identifying the contribution of specific NHE isoforms to macropinocytosis.

Underscoring the importance of NHE-dependent macropinocytosis in tumor growth, EIPA treatment of mice bearing MIA-PaCa2-derived xenograft tumors showed a suppression of intratumoral macropinocytosis that was accompanied by a reduction in tumor size relative to control mice (10). Moreover, EIPA treatment was effective only in tumors with high macropinocytic activity, as tumor growth rate was not affected in tumors derived from BxPC3 cells, which display low levels of macropinocytosis. These results indicated that enhanced macropinocytic activity in particular tumors might represent a metabolic vulnerability that can be specifically harnessed to restrain tumor growth.

In addition to nascent macropinosome formation, later stages of macropinosome maturation where the degradation of the macropinocytosed cargo occurs are also dependent on pH regulation. Macropinosome maturation includes fusion with lysosomes, which facilitates the delivery of the machinery necessary for compartmental acidification and the lysosomal proteases that are responsible for protein catabolism (41). Vacuolar H^+^-ATPases (V-ATPases) function to maintain the acidic pH of different intracellular organelles such as late endosomes and lysosomes and these proton pumps play a critical role in vesicular trafficking and protein degradation (51). Concordantly, inhibition of V-ATPases by bafilomycin A1 has been shown to impair downstream events in the macropinocytosis pathway (Figure 1). For instance, bafilomycin A1 inhibits degradation of LDL causing its accumulation in macropinocytic vesicles in macrophages (52). Furthermore, protein degradation of macropinocytosed albumin was prevented by treatment with bafilomycin A1 in *KRAS*-mutant PDAC cells (10). Interestingly, bafilomycin A1 also inhibits the initial stages of macropinocytosis in *HRAS*-mutant T24 bladder cells (53), as well as in *KRAS*-mutant A549 lung cells (54). These effects of bafilomycin on nascent macropinosome formation may be a result of either (1) perturbations of cytosolic pH due to impaired pumping of protons into lysosomes or (2) alterations of submembranous pH due to inhibition of proton pumping to the extracellular space. Supporting the second possibility, several studies have demonstrated that V-ATPases can reside at the plasma membrane in *KRAS*-mutant PDAC and breast tumor cells and contribute to pH regulation at the plasma membrane (55, 56).

Altogether, these results suggest that pH homeostasis, both at the cellular and organellular level, is vital to properly execute the macropinocytosis program. Therefore, from the perspective of the pathological state, pH homeostasis is at the center of cancer cell metabolism and is critical to the role of macropinocytosis as a vital nutrient supply route that supports cancer cell growth and survival.

## Macropinocytosis as a Survival Strategy in Tumors

Oncogenic Ras triggers a myriad of cellular adaptations to promote the metabolic rewiring that allows cancer cells to sustain unrestrained proliferation [reviewed in Ref. (57)]. Such rewiring includes enhanced glucose uptake and glycolytic activity, shifts in glutamine metabolism and redox balance (58, 59), increased flux of glucose to anabolic pathways such as hexosamines and ribose-5-phosphate (60), as well as upregulation of the major nutrient-scavenging mechanisms: autophagy (61–63) and macropinocytosis (10). It was recently demonstrated that extracellular amino acids and lipids rather than glucose contribute to the majority of cell biomass in proliferating cancer cells (64). These findings underscore the relevance of nutrient-scavenging pathway exploitation by cancer cells to support tumor growth and survival.

Unlike autophagy, which generates nutrients from a limited supply of intracellular organelles and cytosolic proteins, macropinocytosis serves as a feeding mechanism by internalizing and degrading extracellular proteins. In this way, the resulting protein-derived amino acids can be utilized by the tumor to fuel central carbon metabolism, in addition to other metabolic pathways. This function has been proven to be particularly relevant to sustaining tumor growth in nutrient-deprived environments both *in vitro* and *in vivo*. For instance, mutant *KRAS*-driven pancreatic cancer cells, which rely on glutamine metabolism to support their growth, can maintain proliferation under glutamine-limiting conditions if supplied with extracellular serum albumin. Importantly, this escape from the deleterious effects of glutamine deprivation is suppressed by the inhibition of macropinocytosis (10). Similarly, extracellular serum albumin has the ability to reverse a proliferation arrest in PDAC cells grown in the absence of essential amino acids, such as leucine (11, 12). Tracing experiments with labeled extracellular protein showed that protein-derived amino acids are indeed incorporated into TCA cycle metabolites, supporting the growth-promoting role of macropinocytosis in Ras-transformed cells (10). Thus, macropinocytosis is necessary to support cell growth under nutrient-deprived conditions. Whether the extent of macropinocytosis can be dialed up or down depending on nutritional status remains to be elucidated.

The potential importance of macropinocytosis in human cancer is underscored by the observation that PDAC tumor tissues from Whipple procedure patients display enhanced macropinocytic uptake relative to adjacent non-neoplastic regions (11). Such PDAC tumors are hypovascularized and are depleted of amino acids (11); therefore, extracellular protein scavenging represents an attractive alternative for nutrient acquisition in these tumors as opposed to the import of free, circulating amino acids, which would depend on adequate perfusion. In agreement with this, extracellular protein uptake and catabolism *via* macropinocytosis has been directly evidenced *in vivo* in murine PDAC tumors (28). Moreover, inhibition of macropinocytosis *via* EIPA treatment suppressed tumor growth in xenograft tumors (10). Although the majority of the studies on the utilization of extracellular protein as a nutrient source have been performed in pancreatic cancer cells and animal models, similar results have also been reported in Ras-driven cancer cells of different tissue origins, such as bladder, lung, sarcoma, and colon cancer (10, 31, 65). The cell growth effects mediated by macropinocytosis under glutamine-deprived conditions were suppressed in *KRAS*-mutant lung, sarcoma, and colon cells, when the Rac1 activator Dock1 was inhibited either pharmacologically or by genetic ablation. Moreover, treatment with a Dock1 inhibitor decreased tumor growth and metastasis in mice (31). In addition to tumor cells, macropinocytosis was also observed in the stromal compartment of PDAC tumors (11). Further studies are necessary to evaluate the potential contribution of macropinocytosis to survival and growth of these cells.

The intracellular degradation of extracellular proteins acquired *via* macropinocytosis is dependent upon lysosomes, which are tightly controlled by the mammalian target of rapamycin complex (mTORC1), a key regulator of cell growth that responds to nutrient availability (66). Lysosomal catabolism of extracellular proteins has been shown to activate mTORC1 in Ras-transformed cells (12), and furthermore, macropinocytosis-derived lysosomal amino acids are required for rapid activation of mTORC1 in response to growth factor stimulation to promote cell growth (67). On the other hand, mTORC1 activation seems to minimize lysosomal degradation of macropinocytosed proteins. Suppression of mTORC1 by rapamycin or torin1 enhances protein catabolism and proliferation in amino acid-starved PDAC cells *in vitro*, and in hypovascularized, nutrient-poor regions of PDAC tumors (12). Thus, although it seems that mTORC1 does not directly regulate the early steps of macropinocytosis, it does play a pivotal role in regulating the degradation of proteins that are internalized by macropinosomes, allowing for the coordination of protein catabolism in response to nutrient availability. Hence, when free amino acids are plentiful, mTORC1 activation could suppress the catabolism of macropinocytosed proteins and conversely, as amino acid levels decrease upon consumption, mTORC1 suppression could allow for enhanced protein degradation. It is also conceivable that as protein-derived amino acids are produced, they, in turn, activate mTORC1, establishing a feedback regulatory loop that serves to shift between nutrient acquisition pathways depending on nutrient availability (Figure 1).

Finally, molecules other than serum albumin are also internalized *via* macropinocytosis, and uptake of these molecules might also contribute to tumor metabolism and growth. For example, tumor cells can internalize and catabolize extracellular matrix molecules, such as fibronectin and collagen (28, 68). Like serum albumin, internalization of these matrix molecules serves to produce protein-derived amino acids that can support tumor cell growth. In addition to extracellular proteins, *KRAS*-mutant A549 lung cancer cells can also macropinocytose extracellular ATP in order to increase the intracellular ATP pool (69). Ras-driven cancer cells are also known to have increased lipid scavenging to support tumor metabolism (70, 71), and although the mechanisms remain to be elucidated, it is reasonable to hypothesize that serum lipids bound to albumin are taken up *via* macropinocytosis.

## Concluding Remarks

The critical role that metabolic reprogramming and adaptation strategies play in supporting the growth of tumors is now widely recognized. By inducing the internalization of extracellular proteins, and other macromolecules, that can be further processed and utilized to fuel different metabolic pathways, macropinocytosis provides not only a survival mechanism under nutrient-scarce conditions but also the potential for unrestricted tumor growth in an adverse tumor microenvironment. For this reason, targeting macropinocytosis has emerged as a novel therapeutic strategy that requires further investigation. Understanding the molecular events that drive macropinocytosis in the context of different cancers might inform the design of more specific and potent inhibitors. Given that macropinocytosis is crucial to sustaining tumor growth under nutrient-deprived conditions, it is possible that macropinocytosis inhibition would be particularly beneficial in patients suffering from severely hypoxic or hypovascularized tumors, such as PDAC. Moreover, studies focused on combination therapies employing macropinocytosis inhibitors in conjunction with other metabolic pathway inhibitors could pave the way for improved therapeutic outcomes.

## Author Contributions

MR and CC conceived, organized, and wrote the manuscript.

## Conflict of Interest Statement

The authors declare that the research was conducted in the absence of any commercial or financial relationships that could be construed as a potential conflict of interest.
